# Tolerability of linezolid in patients with drug-resistant TB in Daru, Western Province, PNG

**DOI:** 10.5588/pha.24.0041

**Published:** 2024-12-01

**Authors:** M. Boga, T. Marukutira, A. Murray, G.G. Chan, G.K. Huang

**Affiliations:** ^1^ Western Provincial Health Authority, Daru, Western Province, Papua New Guinea (PNG);; ^2^ Burnet Institute, Daru, Western Province, PNG;; ^3^ Monash University, Melbourne, VIC, Australia;; ^4^ Burnet Institute, Melbourne, VIC, Australia;; ^5^ University of Melbourne, Melbourne, VIC, Australia.

**Keywords:** Papua New Guinea, adverse events, anaemia, myelosuppression

## Abstract

**SETTING:**

Linezolid (LZD) has emerged as a critical agent and core component of regimens to treat drug-resistant TB (DR-TB); however, there are ongoing uncertainties regarding its safety and the optimal dosing approach. Since 2016, linezolid has been used in the treatment of patients diagnosed with DR-TB at Daru Provincial Hospital, PNG.

**OBJECTIVE:**

To describe the patient characteristics, treatment outcomes, and tolerability of linezolid-containing regimens used to treat DR-TB in Daru, Western Province.

**DESIGN:**

This was a retrospective analysis of programmatic data.

**RESULTS:**

A total of 632 eligible patients were diagnosed with DR-TB during the study period. Of these, 580 (91.8%) were commenced on a LZD-containing regimen. Among patients with baseline haemoglobin results available (380/632, 60.1%), the median value was 10.1 g/dL, with a mean corpuscular volume of 70.7 fL, 78.1% of those with haemoglobin results were anaemic at baseline. Despite this, 242/580 (41.7%) patients were able to complete the full LZD course of treatment (typically 18 months) without dose reduction or interruption. Treatment success was observed in 87.9% of the cohort. Neurotoxicity was not commonly reported, but adverse events were likely under-reported.

**CONCLUSION:**

In this cohort, with high rates of baseline anaemia, prolonged therapy with LZD was relatively well tolerated.

## Abstract (fr)

**CONTEXTE:**

Le linézolide (LZD) est devenu un agent critique et un composant essentiel des régimes de traitement de la TB pharmacorésistante (DR-TB), mais des incertitudes subsistent quant à son innocuité et à l'approche posologique optimale. Depuis 2016, le LZD est utilisé dans le traitement des patients diagnostiqués avec la DR-TB à l'hôpital provincial de Daru, en Papouasie-Nouvelle-Guinée.

**OBJECTIF:**

Décrire les caractéristiques des patients, les résultats du traitement et la tolérabilité des schémas thérapeutiques contenant du LZD utilisés pour traiter la TB pharmacorésistante à Daru, dans la province de l'Ouest.

**CONCEPTION:**

Il s'agissait d'une analyse rétrospective des données programmatiques.

**RÉSULTATS:**

Au total, 632 patients éligibles ont été diagnostiqués avec la DR-TB au cours de la période d'étude. De ce nombre, 580 (91,8%) ont été administrés sous un régime contenant des LZD. Parmi les patients pour lesquels des résultats d'hémoglobine de base étaient disponibles (380/632, 60,1%), la valeur médiane était de 10,1 g/dL, avec un volume globulaire moyen de 70,7 fL, et 78,1% de ceux dont les résultats d'hémoglobine étaient anémiques au départ. Malgré cela, 242/580 (41,7%) patients ont été en mesure de terminer le traitement complet de la LZD (généralement 18 mois) sans réduction ou interruption de dose. Le succès du traitement a été observé chez 87,9% de la cohorte. La neurotoxicité n'a pas été fréquemment signalée, mais les événements indésirables ont probablement été sous-déclarés.

**CONCLUSION:**

Dans cette cohorte, avec des taux élevés d'anémie de base, le traitement prolongé par LZD a été relativement bien toléré.

In recent years, linezolid (LZD) has emerged as an important antimicrobial agent in the treatment of patients with multidrug-resistant or rifampicin-resistant TB (MDR/RR-TB).^1^ It is one of three ‘group A’ agents, second-line drugs with the strongest evidence for efficacy and a core component of currently recommended short, all-oral regimens. Emerging evidence on the effectiveness of these regimens has led to their inclusion in WHO guidelines.^2–4^ However, there are ongoing concerns regarding the safety and tolerability of LZD.

Documented risks associated with LZD use include myelosuppression and neuropathy (peripheral, optic).^5^ In a recent trial of the BPaL regimen in adults, the use of LZD at a daily dose of 1,200 mg was linked to frequent adverse events: 81% of patients reported peripheral neuropathy, and nearly half experienced anaemia or thrombocytopenia.^3^ As a result, most patients required a dose reduction or interruption during treatment, with only 15% completing the 26-week treatment without interruption or reduction. Some studies have included baseline anaemia as an exclusion criterion for initiating LZD therapy.^3^

LZD has been used in Papua New Guinea (PNG) for some time and has been incorporated into national protocols for adults and children with MDR/RR-TB.^6^ The standardised ‘long regimen’ typically lasts 18 months and is constructed as per WHO guidance.^1^ Anaemia is commonly reported in PNG populations, mainly due to malaria or pregnancy,^7,8^ but there are no published data on the prevalence of anaemia in patients with TB. The mechanisms of anaemia vary between populations, and LZD may be tolerated by most children and adults with TB, including those with baseline anaemia.

Currently, there are no published data on the use of LZD to treat DR-TB in PNG. Additionally, the association of anaemia with TB and the potential negative impact of LZD on haematological parameters are important knowledge gaps. We aimed to describe the tolerability of LZD-containing regimens used under programmatic conditions for the treatment of patients in Daru, Western Province, PNG.

## METHODS

### Study setting

PNG is a high-burden country for TB and MDR/RR-TB.^9^ Western Province shares international borders with Indonesia and Australia and is divided into four districts: North Fly, Middle Fly, Delta Fly, and South Fly. Daru Island is the provincial capital and the location of Daru Provincial Hospital, which houses the TB Basic Management Unit (BMU) for South Fly District, one of six BMUs in the province. Daru has a population of approximately 20,000 people, with very high annual case notifications of TB and MDR/RR-TB.^10,11^

### Study design and population

We conducted a retrospective descriptive analysis using routine programmatic data for patients of all ages who initiated treatment for DR-TB at the Daru Provincial Hospital BMU between 1 January 2016 and 31 December 2021. Diagnosis was based on bacteriological confirmation with Xpert^®^ MTB/RIF (Cepheid, Sunnyvale, CA, USA) and/or culture, as well as clinical diagnosis. Phenotypic drug susceptibility testing (DST) for LZD was unavailable until 2022 and required samples to be sent to a supranational reference laboratory in Australia.

### Treatment and monitoring

Daru Provincial Hospital BMU is the primary referral TB treatment centre for South, Delta, and Middle Fly Districts, with the capacity to diagnose and manage both drug-susceptible and DR-TB. It has separate wards for inpatient TB care, with a combined bed capacity of 39 beds. The hospital has a TB diagnostic centre, patient counselling services to support patients throughout their treatment, and community-based ‘Daru Accelerated Response for TB’ (DART) sites that provide patient-centred care for people with TB.

All TB patient data are routinely entered into a standard paper-based BMU register and an electronic medical record system (EMRS) (Bahmni v.0.87; Thoughtworks, Chicago, IL, USA). Patients are monitored throughout their treatment as previously described, which includes active drug safety monitoring (aDSM), implemented in Daru in 2016 as part of the rollout of bedaquiline and delamanid in PNG.^10,12^ LZD was introduced into the standardised regimen for MDR/RR-TB in PNG in 2017. Before this, use was individualised based on DST results, patient factors, and clinician preference. LZD dosing followed the 2017 PNG guidelines,^6^ with a standard dose of 600 mg once daily for adults >30 kg and weight-based dosing for children (approximately 10 mg/kg twice daily or 300 mg daily for children >10 years).

Current recommendations include baseline evaluation and monthly monitoring of haemoglobin and platelets while on LZD; however, this is not always feasible due to patient transport issues and interruptions in laboratory services. The monthly clinical review also includes screening for optic neuropathy (using Ishihara charts and visual acuity assessments) and examination for peripheral neuropathy; the frequency of review may be reduced toward the end of treatment. Additional investigations were conducted as per the PNG programmatic management of drug-resistant TB (PMDT) standard operating procedure.^6^ Adverse events were not routinely graded, and recording may depend on the clinician.

In cases of toxicity, dose reduction (e.g. from 600 mg to 300 mg in adults) was permitted at the clinician’s discretion, in line with WHO guidelines.^1,2^ In cases of severe toxicity, LZD could be discontinued, and an alternative agent used. The management of anaemia was determined by the clinician and could include anti-helminth treatment, oral iron supplementation, nutritional supplementation, or blood transfusion.

### Data variables, analysis and statistics

Data from the EMRS were extracted into flat files in a delimited format using scripted SQL queries. Variables extracted included demographic profiles, baseline and follow-up haematological and biochemical indices, duration of treatment on LZD-containing regimens, LZD dosage at initiation and whether it was reduced during treatment, any recorded treatment interruptions and their reasons, and treatment outcomes (favourable or unfavourable). Patient treatment data included dosage, start date, stop date, frequency of administration, and route of administration for each TB drug prescribed during the treatment episode. Data analysis and cleaning were performed using Stata SE v17 (Stata Corp, College Station, TX, USA) and data visualisation using R v4.2.2 (R Computing, Vienna, Austria).

Treatment baseline laboratory results were defined as the earliest laboratory result within 14 days before or after the start of the treatment episode. In some cases, LZD was initiated after the treatment episode began; this decision was made at the clinician’s discretion. Potential reasons for this included severe baseline anaemia corrected before LZD initiation or initiation following receipt of phenotypic DST results. For this reason, LZD baseline results were defined separately from treatment baseline results, as the laboratory result closest to the baseline date within 14 days before or after LZD initiation. The lowest value was taken as the baseline if a patient had multiple results on the same day.

Normal ranges for laboratory tests were provided by the laboratory (Supplementary Data A). The potential impact of LZD on haemoglobin or platelets was identified by the nadir, which was the lowest value recorded in the EMRS for a particular patient at any point during their treatment episode after starting LZD.

Treatment outcomes were defined programmatically and were largely consistent with WHO definitions,^13^ with a slight modification as previously described.^10^ Patients who had a permanent regimen change of two or more anti-TB drugs due to adverse drug reactions were not classified as treatment failures if they were responding well to treatment.

### Ethics approval

Ethics approval was granted by the PNG Medical Research Advisory Council, Port Moresby, PNG.

## RESULTS

A total of 638 patients commenced treatment for MDR-TB between 1 January 2016 and 31 December 2021. Six patients enrolled in 2016 had no data on prescribed drugs for their treatment episode recorded and were excluded from further analysis. Of the 632 patients with data on prescribed drugs, 518 (82%) were bacteriologically confirmed, and 594 (94%) were HIV-negative. A total of 580 (91.8%) patients were recorded as being treated with a regimen that included LZD. Table 1 shows the characteristics of those who received LZD and those who did not. For patients receiving LZD, the median age at treatment initiation was 26 years, the median BMI was 17.5, and 21% were children (<15 years). The proportion of patients who started on LZD-containing regimens increased in 2017 following revisions to national treatment guidelines. Treatment success was reported in 87.9% of patients who received an LZD-containing regimen, compared to 80.8% for those treated with non-LZD regimens. Treatment success for children <14 years was 91.9%.

**TABLE 1.** Characteristics of the cohort that commenced treatment for drug-resistant TB at DPH from 2016 to end 2021 by inclusion of LZD in regimen.

		Drug-o-gram with no LZD	Drug-o-gram with LZD	Total
		(*n* = 52) *n* (%)	(*n* = 580) *n* (%)	(*n* = 632) *n* (%)
Sex	Female	30 (57.7)	277 (47.8)	307 (48.6)
	Male	22 (42.3)	303 (52.2)	325 (51.4)
Age at treatment start, years		23.5 [16.1–33.8]	26.0 [17.0–40.0]	25.7 [17.0–40.0]
Age group at treatment start, years	≥15	41 (78.8)	456 (78.6)	497 (78.6)
	5–14	4 (7.7)	82 (14.1)	86 (13.6)
	<5	7 (13.5)	42 (7.2)	49 (7.8)
Year started treatment	2016	38 (73.1)	73 (12.6)	111 (17.6)
	2017	6 (11.5)	96 (16.6)	102 (16.1)
	2018	5 (9.6)	110 (19.0)	115 (18.2)
	2019	2 (3.8)	123 (21.2)	125 (19.8)
	2020	0 (0.0)	102 (17.6)	102 (16.1)
	2021	1 (1.9)	76 (13.1)	77 (12.2)
Treatment duration, days, median [IQR]			562 [526–609]	562 [526–609]
HIV status	Negative	49 (94.2)	545 (94.0)	594 (94.0)
	Positive	2 (3.8)	18 (3.1)	20 (3.2)
	Unknown	1 (1.9)	17 (2.9)	18 (2.8)
Baseline diagnosis confirmation	Bacteriologically confirmed	44 (84.6)	474 (81.9)	518 (82.1)
	Clinically confirmed	8 (15.4)	105 (18.1)	113 (17.9)
Registration category*	New	32 (61.5)	377 (65.0)	409 (64.7)
	Relapse	8 (15.4)	130 (22.4)	138 (21.8)
	Treatment after failure	11 (21.2)	52 (9.0)	63 (10.0)
	Other	1 (1.9)	21 (3.6)	22 (3.5)
Latest resistance profile^†^	RR-TB	17 (32.7)	218 (37.6)	235 (37.2)
	MDR-TB	32 (61.5)	303 (52.2)	335 (53.0)
	Pre-XDR/XDR-TB	1 (1.9)	46 (7.9)	47 (7.4)
	Other	2 (3.8)	0 (0.0)	2 (0.3)
	Not recorded	0 (0.0)	13 (2.2)	13 (2.1)
Baseline BMI, kg/m^2^, median [IQR]		17.2 [14.5–19.5]	17.5 [15.5–19.6]	17.5 [15.4–19.6]
Outcome	Completed	30 (57.7)	374 (64.5)	404 (63.9)
	Cured	12 (23.1)	136 (23.4)	148 (23.4)
	Died	6 (11.5)	41 (7.1)	47 (7.4)
	Failed	0 (0.0)	4 (0.7)	4 (0.6)
	LTFU	3 (5.8)	21 (3.6)	24 (3.8)
	Not evaluated	1 (1.9)	4 (0.7)	5 (0.8)
Favourable outcome (cured or completed)		42 (80.8)	510 (87.9)	552 (87.3)

* Includes 'Other' consists of 'Treatment after LTFU', 'Other previously treated', and 'Not recorded'.

† Includes 'Other' and consists of mono and polydrug-resistant cases.

DPH = Daru Provincial Hospital; LZD = linezolid; IQR = interquartile range; RR-TB = rifampicin-resistant TB; MDR-TB = multidrug-resistant TB; XDR-TB = extensively drug-resistant TB; BMI = body mass index; LTFU = loss of follow-up.

**FIGURE 1.**
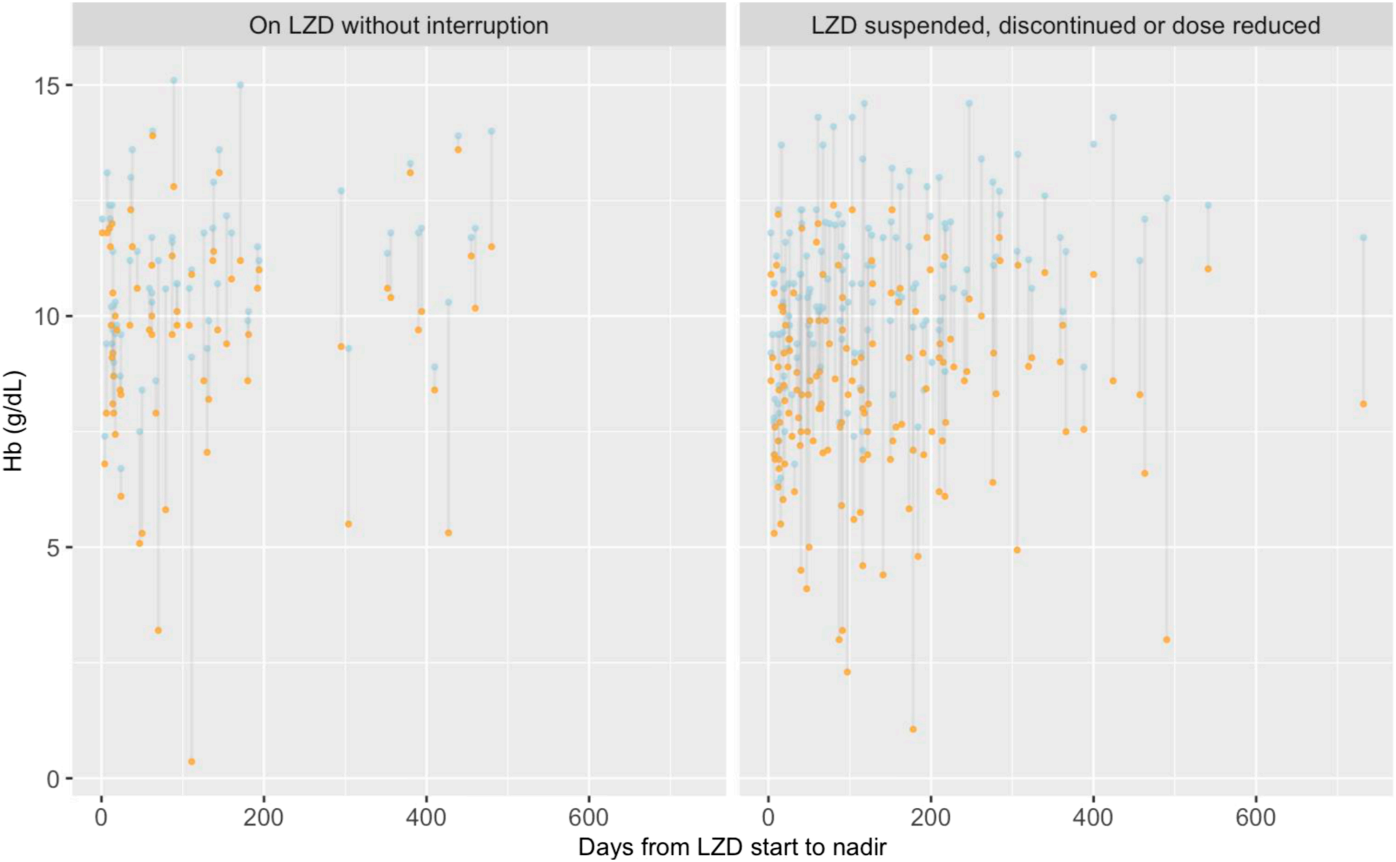
Time to onset of Hb nadir and magnitude of nadir. Baseline values are indicated in blue and nadir values in orange. Each line represents an individual patient. Hb = haemoglobin; LZD = linezolid.

**FIGURE 2.**
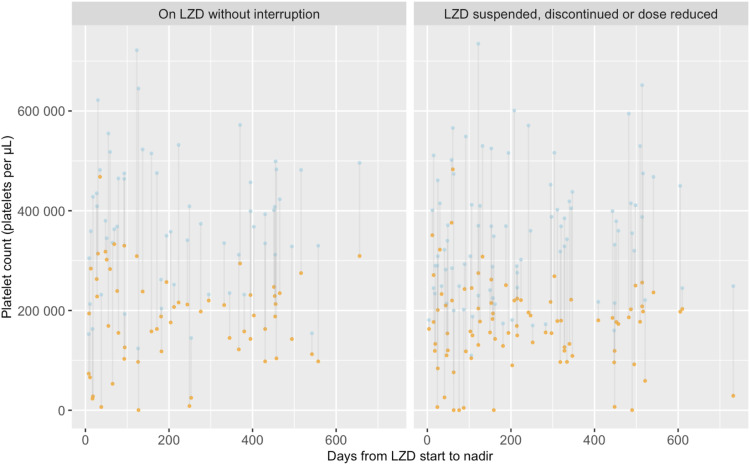
Time to onset of platelet nadir and magnitude of nadir. Baseline values are indicated in blue and nadir values in orange. Each line represents an individual patient. LZD = linezolid.

Median baseline laboratory values are reported in Table 2 with disaggregation by gender and age; however, baseline results were not available for all patients. Baseline haemoglobin was available in 380 (60.1%) patients, with a median of 10.1 g/dL and a mean corpuscular volume of 71 fL. Of these, 297/380 (78.1%) were anaemic at baseline. Among adult females, the median haemoglobin was 9.2 g/dL. There were 369 patients with additional haemoglobin results after LZD initiation, and in 218 patients, haemoglobin was lower than baseline; the median nadir was 9.4 g/dL. The median platelet count at baseline was 350,000 mcL in 172 patients, and the median nadir was 177,000 mcL in 147 patients with further readings, where the nadir was not the baseline. The time to haemoglobin and platelet nadirs and the magnitude of reduction are charted for individual patients in Figures 1 and 2. Median days to haemoglobin and platelet nadirs were respectively95.5 (interquartile range [IQR] 30–191.5) and 195 (IQR 68–391). Of the 66 patients with a normal or high haemoglobin at the time of LZD initiation, 51 (77.3%) recorded a nadir in the low or critically low range, and 143 with a normal or high platelet count at LZD initiation, 59 (67%) recorded a nadir in the low or critically low range.

**TABLE 2.** Laboratory baseline values disaggregated by age, sex and whether or not receiving a LZD-containing regimen.

Treatment baseline result	Baseline results available	Not on LZD, median [IQR]				On LZD, median [IQR]				Total, median [IQR]
		Female		Male		Female		Male		
		Age <15 years	Age ≥15 years	Age <15 years	Age ≥15 years	Age <15 years	Age ≥15 years	Age <15 years	Age ≥15 years	
		(*n* = 8)*	(*n* = 22)	(*n* = 3)*	(*n* = 19)	(*n* = 64)	(*n* = 213)	(*n* = 60)	(*n* = 243)	(*n* = 632)
Haemoglobin	380		9.8 [7.6–11.6]		10.3 [9.17–10.7]	10 [8.7–10.7]	9.2 [7.9–10.6]	9.8 [9.1–10.6]	10.99 [9.6–12.1]	10.1 [8.665–11.45]
MCV	175		65.3 [63–66.7]		73.5 [61.1–76.9]	65.45 [61.9–71.6]	71.7 [67.8–76.9]	65.4 [60.8–68.9]	73.8 [68.1–79.5]	70.7 [65.3–76.9]
BUN	354		2.95 [2.58–3.66]		3.68 [3.57–4.745]	2.75 [2.25–3.39]	3.415 [2.65–4.2]	2.975 [2.245–4.105]	3.8 [2.96–4.675]	3.47 [2.7–4.35]
Creatinine	371		47.5 [37–61]		87 [64–99.5]	30 [18–41]	56 [50–69.5]	32 [25–38]	76 [64–87]	62 [45–77]
Platelets	185		403,500 [229,000–491,000]		357,000 [330,000–604,900]	397,850 [311,500–475,350]	341,000 [235,000–450,000]	385,000 [330,000–532,000]	341,500 [235,000–417,050]	350,000 [249,000–459,000]
ALT	309		17.5 [12–24]		51 [20–81]	15.5 [12–29]	18 [12.5–29.5]	17 [11–24]	21 [14–30]	19 [13–29]
Anaemia, %	380		66.7		80	60	81.3	57.1	89.8	78.1

* For some categories, e.g. Males and Females not on LZD <15 years; although there were patients categorised, none had valid baseline results available.

LZD = linezolid; IQR = interquartile range; MCV = mean cell volume; BUN = blood urea nitrogen; ALT = alanine aminotransferase

Of the 580 patients treated with a LZD-containing regimen, 242 (41.7%) were able to complete treatment without dose reduction or interruption. Table 3 compares the characteristics of this group to the other 338 patients who had LZD reduced, suspended, or discontinued before outcome assignment. Of the 119 (35.3%) patients with LZD permanently discontinued, 36 had a new drug initiated within the following 30-day period. Median age (32.0 years) and treatment success (90.5%) were higher in those who required LZD dose reduction or interruption than those who did not. Adverse drug reactions are reported in Supplementary Data B for the 338 patients with LZD cessation, suspension, or dose reduction where an event was recorded at LZD discontinuation. The most common were peripheral neuropathy (8.9%) and anaemia (5.3%). Two patients (0.6%) were reported to have optic neuritis/neuropathy, although blurred or decreased vision was noted in five (1.5%).

**TABLE 3.** Characteristics of LZD enrolment cohorts with and without LZD interruption and treated at DPH in 2016–2021.

		On LZD without interruption	LZD suspended, discontinued or dose reduced	Total
		(*n* = 242) *n* (%)	(*n* = 338) *n* (%)	(*n* = 580) *n* (%)
Sex	Female	112 (46.3)	165 (48.8)	277 (47.8)
	Male	130 (53.7)	173 (51.2)	303 (52.2)
Age at treatment start, years, median [IQR]		20.1 [11.2–28.7]	32.0 [21.3–45.0]	26.0 [17.0–40.0]
Age group at treatment start, years	≥15	157 (64.9)	299 (88.5)	456 (78.6)
	5–14	55 (22.7)	27 (8.0)	82 (14.1)
	<5	30 (12.4)	12 (3.6)	42 (7.2)
Treatment duration, days, median [IQR]		553.5 [511–607]	566 [537–610]	562 [526–609]
HIV status	Negative	225 (93.0)	320 (94.7)	545 (94.0)
	Positive	5 (2.1)	13 (3.8)	18 (3.1)
	Unknown	12 (5.0)	5 (1.5)	17 (2.9)
Baseline diagnosis confirmation	Bacteriologically confirmed	191 (79.3)	283 (83.7)	474 (81.9)
	Clinically confirmed	50 (20.7)	55 (16.3)	105 (18.1)
Registration category*	New	169 (69.8)	208 (61.5)	377 (65.0)
	Relapse	39 (16.1)	91 (26.9)	130 (22.4)
	Treatment after failure	25 (10.3)	27 (8.0)	52 (9.0)
	Other	9 (3.7)	12 (3.6)	21 (3.6)
Latest resistance profile^†^	RR-TB	81 (33.5)	137 (40.5)	218 (37.6)
	MDR-TB	142 (58.7)	161 (47.6)	303 (52.2)
	Pre-XDR/XDR-TB	12 (5.0)	34 (10.1)	46 (7.9)
	Not recorded	7 (2.9)	6 (1.8)	13 (2.2)
Baseline BMI, kg/m^2^, median [IQR]		17.0 [14.6–19.5]	17.7 [15.8–19.7]	17.5 [15.5–19.6]
Treatment outcome	Completed	160 (66.1)	214 (63.3)	374 (64.5)
	Cured	44 (18.2)	92 (27.2)	136 (23.4)
	Died	23 (9.5)	18 (5.3)	41 (7.1)
	Failed	0 (0.0)	4 (1.2)	4 (0.7)
	LTFU	13 (5.4)	8 (2.4)	21 (3.6)
	Not evaluated	2 (0.8)	2 (0.6)	4 (0.7)
Successful treatment outcome		204 (84.3)	306 (90.5)	510 (87.9)
Had LZD dose reduced			256 (75.7)	256 (44.1)
Had LZD suspended			113 (33.4)	113 (19.5)
Had LZD discontinued			119 (35.3)	119 (20.5)

* Includes 'Other' consists of 'Treatment after LTFU', 'Other previously treated', and 'Not recorded'.

† Includes 'Other' and consists of mono and polydrug-resistant cases.

DPH = Daru Provincial Hospital; LZD = linezolid; IQR = interquartile range; RR-TB = rifampicin-resistant TB; MDR-TB = multidrug-resistant TB; XDR-TB = extensively drug-resistant TB; BMI = body mass index; LTFU = loss of follow-up.

## DISCUSSION

These novel findings from PNG in a large cohort of patients treated for MDR-TB with LZD-containing regimens show high rates of treatment success that exceed the targets set in the PNG National Strategic Plan.^14^ It is noteworthy that the treatment success rate was significantly higher among those who experienced dose reduction, suspension, or treatment interruption compared to those who completed treatment without interruption. However, numerous potential confounders may account for these results, including temporal changes in background regimens and programmatic support. It is also plausible that patients with dosing changes were followed more closely and better engaged in care.

This is the first study to report on the prevalence of anaemia in people with TB in PNG. Microcytic anaemia was common across all ages and genders, with adult females having the lowest median haemoglobin values, but the causes remain unclear. Possible underlying causes include thalassaemia, which has been described in various settings in PNG,^15^ iron deficiency due to malnutrition or hookworm infestation, or anaemia of chronic disease related to TB. In some programmatic settings^16^ and trials,^3^ baseline anaemia has been an exclusion criterion for initiating an LZD-containing regimen, but not in others.^4^ Currently, baseline anaemia is not listed as a specific exclusion criterion in the PNG PMDT guidelines,^6^ and management is at the clinician's discretion. Effective management of baseline anaemia may help mitigate the potential impact of LZD on myelosuppression.

The strength of our study lies in its execution under routine programmatic conditions, reflecting real-world experience in a resource-constrained setting. The major limitation of our study was the lack of data available to determine LZD-related tolerability and toxicity. Haemoglobin or platelet count data were unavailable at baseline for a number of patients and were not recorded during follow-up for the majority. Although many patients had LZD stopped, interrupted, or reduced, few adverse events were reported, making it impossible to determine the actual frequency of events such as peripheral or optic neuropathy. Resource limitations in this setting, including prolonged stock-outs of laboratory reagents or limited stock, led to prioritisation of testing for the most unwell patients. There were also changes to facility-based follow-up and care during the COVID pandemic. While the nadir was chosen as a pragmatic variable, it does not fully capture the nuances of assessing myelotoxicity, and anaemia during treatment may have other causes. The management of side effects in patients on LZD was not routinely recorded.

Despite these limitations, the use of LZD was not observed to be associated with marked myelosuppression. A recent individual patient data (IPD) meta-analysis by Hasan et al.^17^ on the safety and tolerability of LZD in clinical trials noted that myelosuppression was relatively uncommon when lower doses of LZD were used (600 mg daily for adults, compared to 1,200 mg daily). In addition, permanent discontinuation of LZD was rare (<5%) when lower doses were used. This compares to a permanent discontinuation rate of 35.3% in our study. A possible reason for this discrepancy is that treatment duration in our study was much longer, typically 18 months, than 6–9 months in the IPD.

Further research is needed to determine the optimal schedule for active drug safety monitoring, given the relatively modest myelosuppression observed with current dosing strategies. Similarly, the optimal dosing approach for LZD remains unknown and may vary between individuals due to pharmacokinetic factors. Therapeutic drug monitoring may have potential but is largely inaccessible in most resource-limited settings. As shorter regimens containing LZD are introduced, it will be important to prospectively collect data to determine the incidence and severity of adverse events, particularly those related to dosing.

In conclusion, despite high rates of baseline anaemia and long treatment durations, LZD-containing regimens for MDR-TB were relatively well tolerated and effective in the PNG context. This study provides important baseline data for assessing the introduction of shorter regimens for DR-TB. Active drug safety monitoring remains crucial, and the reporting of adverse events needs improvement.

## Supplementary Material


