# Supplementary material for: Tolerability of linezolid in patients with drug-resistant TB in Daru, Western Province, PNG
Source: Public Health Action. 2024 Dec 1;14(4):158–63. doi: 10.5588/pha.24.0041 (PMC11604151; doi:10.5588/pha.24.0041)
Supplement: Supplementary file 1 [file pha24-0041_supplementarydata1.pdf]

http://dx.doi.org/10.5588/pha.24.0041

# Tolerability of linezolid in patients with drug-resistant TB in Daru, Western Province, PNG

## SUPPLEMENTARY DATA

### A: Haematological and biochemical profiles with normal and abnormal ranges

| Laboratory profile | | Children (<15 yrs) | Adult Male | Adult Female |
|---|---|---|---|---|
| Haemoglobin g/dl | Low | <10.2 | <12.55 | <11.59 |
| | Normal | 10.2-13.4 | 12.55-16.99 | 11.59-15.11 |
| | Elevated | >13.4 | >16.99 | >15.11 |
| Platelets /ml | Low | <189 | <146.5 | <169.1 |
| | Normal | 189-403 | 146.5-351.5 | 169.1-368.3 |
| | Elevated | >403 | >351.5 | >368.3 |
| Mean Corpuscular Volume (MCV) | Low | <71.3 | <78.3 | <80.0 |
| | Normal | 71.3-87.6 | 78.3-95.5 | 80.0-98.0 |
| | Elevated | >87.6 | >95.5 | >98.0 |
| Mean Corpuscular Haemoglobin Concentration (MCHC) | Low | <31.8 | <33.0 | <32.9 |
| | Normal | 31.8-35.8 | 33.0-35.3 | 32.9-35.4 |
| | Elevated | >35.8 | >35.3 | >35.4 |
| Creatinine umol/l | Low | <53 | <53 | <35 |
| | Normal | 53-97 | 53-97 | 35-71 |
| | Elevated | >97 | >97 | >71 |

| | | | | |
|---|---|---|---|---|
| Urea | Low | <2.86 | <2.86 | <2.86 |
| | Normal | 2.86-8.21 | 2.86-8.21 | 2.86-8.21 |
| | Elevated | >8.21 | >8.21 | >8.21 |
| Alanine transaminase (ALT) (GPT) | Low | <4 | <4 | <4 |
| | Normal | 4-44 | 4-44 | 4-44 |
| | Elevated | >44 | >44 | >44 |
| Blood sugar level | Low | <3.9 | <3.9 | <3.9 |
| | Normal | 3.9-6.1 | 3.9-6.1 | 3.9-6.1 |
| | Elevated | >6.1 | >6.1 | >6.1 |

*Manning L, Laman M, Townsend MA, Chubb SP, Siba PM, Mueller I, Davis TM. Reference intervals for common laboratory tests in Melanesian children. Am J Trop Med Hyg. 2011 Jul;85(1):50-4. doi: 10.4269/ajtmh.2011.11-0095. PMID: 21734123; PMCID: PMC3122342.*

**B: Adverse events reported at linezolid stop among patients with linezolid cessation, suspension, or dose reduction (for enrolment cohorts commencing 2016-2021)**

| | **Total N=338** |
|---|---|
| **Adverse event** | **N (%)** |
| Anaemia | 18 (5.3%) |
| Thrombocytopenia | 5 (1.5%) |
| Blurred or decreased vision | 11 (3.3%) |
| Peripheral neuropathy | 30 (8.9%) |
| | |
| Optic neuritis | 2 (0.6%) |
| Vomiting | 2 (0.6%) |
| Hearing | 1 (0.3%) |
| Ototoxicity | 1 (0.3%) |
| Toxicity due to injectables | 1 (0.3%) |
| Toxicity (unspecified) | 1 (0.3%) |
| Hepatitis | 1 (0.3%) |
| Decreased heart rate | 1 (0.3%) |
